# Clinical Outcomes of Ankaferd Hemostat Mouthwash for High-Grade Oral Mucositis During Systemic Anticancer Therapy: A Single-Center, Retrospective, Single-Arm Cohort Study

**DOI:** 10.3390/healthcare14183005

**Published:** 2026-09-14

**Authors:** Gokhan Colak, Bilgin Demir, Merve Turan, Esin Oktay

**Affiliations:** Department of Medical Oncology, Faculty of Medicine, Aydin Adnan Menderes University, 09100 Aydin, Turkey; drgokhancolak@gmail.com (G.C.); drmerveturan@gmail.com (M.T.); esinct@gmail.com (E.O.)

**Keywords:** oral mucositis, ankaferd hemostat, systemic cancer therapy, pain, mucosal healing

## Abstract

**Highlights:**

**What are the main findings?**

- In this retrospective, single-arm cohort of 43 solid tumor patients with high-grade (WHO Grade 3–4) oral mucositis, 86.0% of patients reached WHO Grade 0 during follow-up after Ankaferd Hemostat initiation, with a median time to Grade 0 of 4 days.
- Pain intensity (NRS) decreased from a median of 5 to 0 during the observation period, and no Grade ≥ 2 adverse events were documented.

**What are the implications of the main findings?**

- These real-world observations suggest that Ankaferd Hemostat mouthwash may be a feasible and well-tolerated supportive care option in patients with severe, hemorrhagic oral mucositis, without inference of a causal treatment effect given the single-arm design.

**Abstract:**

**Background/Objectives**: Oral mucositis (OM) is a serious toxicity that develops due to cancer treatment and impairs patients’ quality of life. Ankaferd Hemostat (AH), a phytotherapeutic agent, has anti-inflammatory and tissue-healing properties. This study evaluated the clinical outcomes and safety of topical AH in adult patients with solid tumors who developed grade 3 or 4 OM during systemic anticancer therapy. **Methods**: A single-center retrospective, single-arm study was conducted between 2020 and 2024 and included 43 patients with solid tumors. They developed OM while undergoing systemic cancer therapy and were treated with topic AH. Mucositis severity was graded using the World Health Organization (WHO) OM scale, and pain intensity was assessed using a Numeric Rating Scale (NRS) daily, from baseline until clinical resolution. Treatment response, recovery time and adverse events were analyzed. **Results**: Of the 43 patients included in the study, 81.4% had grade 3 OM and the others had grade 4 OM. Grade 0 was observed in 86% of patients after treatment. Among the 37 patients achieving Grade 0, median time to recovery was 4 days (IQR 4–5). The median NRS score was 5 points before treatment and 0 after treatment (*p* < 0.001). No Grade ≥ 2 adverse events were observed. Only one patient reported mild dry mouth. **Conclusions**: In this retrospective cohort, AH mouthwash was associated with rapid mucosal recovery and no severe adverse events were documented in patients with high-grade OM, supporting its potential to reduce oncological treatment toxicity; prospective controlled studies are warranted to confirm these findings.

## 1. Introduction

Oral mucositis (OM) is one of the most common complications encountered during systemic cancer treatments [1]. The pathophysiology of OM extends beyond direct cytotoxic damage to rapidly dividing basal epithelial cells; it is a complex, multiphase process involving DNA damage, the generation of reactive oxygen species (ROS), and the activation of inflammatory cascades. Cytotoxic agents upregulate pro-inflammatory cytokines, notably tumor necrosis factor-alpha (TNF-α) and interleukin-6 (IL-6), which amplify mucosal damage and lead to extensive ulceration [2,3].

Clinically, OM initially presents as mild mucosal erythema, which subsequently progresses to painful ulcerations and hemorrhage. This progressive epithelial breakdown profoundly impairs oral intake and hygiene, rendering patients highly susceptible to secondary infectious complications [4]. The severity of OM is standardized using the World Health Organization (WHO) grading criteria (grades 0–4) [5]. High-grade mucositis (grades 3 and 4) represents a severe clinical challenge characterized by a profound inability to tolerate oral nutrition. Consequently, these advanced grades often necessitate hospital admission and intensive supportive care, including parenteral nutrition. Most critically, severe OM frequently mandates dose reductions or interruptions in systemic oncologic therapies, thereby compromising both the patients’ quality of life and potentially their survival outcomes [1,6]. Despite its clinical significance, the management of OM remains a major unmet clinical need. According to current Multinational Association of Supportive Care in Cancer and International Society of Oral Oncology (MASCC/ISOO) and ESMO guidelines, available interventions, such as basic oral hygiene, cryotherapy, and topical analgesics, are largely palliative, and therapeutic options proven to accelerate mucosal healing in high-grade OM are remarkably limited [2,7].

Recently, Ankaferd hemostat (AH; also marketed as Ankaferd Blood Stopper^®^, ABS), a herbal-based hemostatic product, has attracted considerable attention in clinical studies in Turkey due to its wound healing and anti-inflammatory effects [8,9]. AH consists of herbal extracts from *Alchemilla vulgaris*, *Glycyrrhiza glabra*, *Thymus vulgaris*, *Urtica dioica* and *Vitis vinifera* [10]. Upon contact with blood or tissue, AH induces the very rapid (<1 s) formation of an encapsulated protein network, primarily through interactions with fibrinogen and other plasma proteins, which achieves structural hemostasis independent of the classical coagulation cascade. This protein matrix concurrently promotes vital erythrocyte aggregation, mediated via spectrin and ankyrin membrane receptors, thereby providing focal points for tissue stabilization and wound sealing [11]. In preclinical animal models, AH-based formulations have been shown to exert anti-inflammatory and fibroproliferative effects that accelerate wound healing [12,13]; antimicrobial and antioxidant properties have also been proposed for related plant-based extracts, although these require further direct confirmation in the context of AH [12,13]. These pleiotropic properties may be particularly relevant to high-grade OM, in which ulceration, mucosal bleeding, inflammatory amplification, microbial colonization, and impaired epithelial repair coexist. Nevertheless, clinical evidence supporting the therapeutic use of AH in established OM remains limited, and previous studies have predominantly involved small cohorts, pediatric populations, hematological malignancies, or prophylactic administration [10,14,15].

This retrospective study aimed to evaluate the clinical outcomes and safety of topical AH in adult patients with solid tumors who developed established grade 3 or 4 OM during systemic anticancer therapy. To our knowledge, this is among the larger reported therapeutic cohorts evaluating AH in established high-grade OM among adults with solid tumors.

## 2. Materials and Methods

### 2.1. Study Design and Patient Population

**Figure 1:**
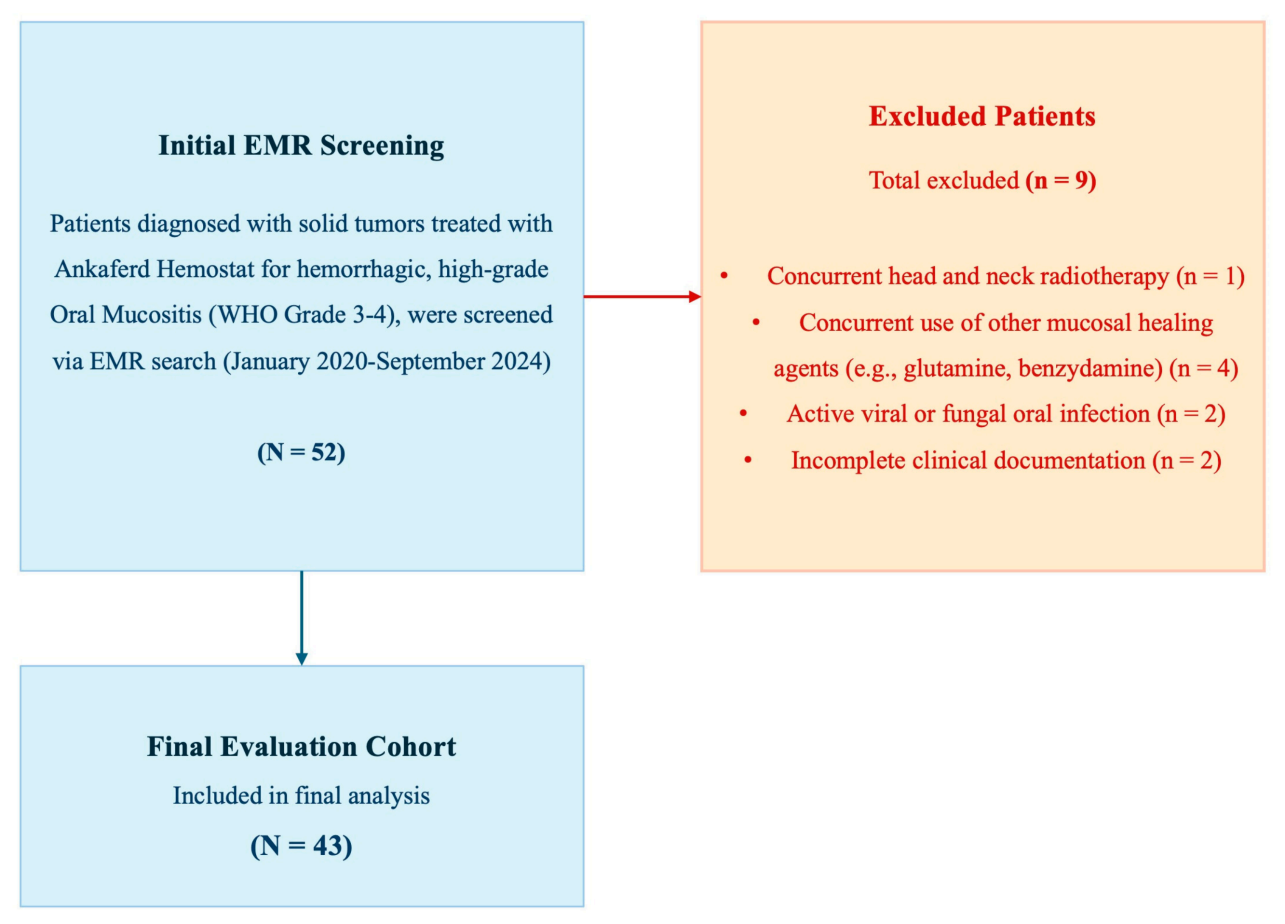
Flow diagram of patient selection. EMR, electronic medical record.

This single-center, retrospective, observational study was conducted at the Department of Medical Oncology, Adnan Menderes University Faculty of Medicine Hospital. We systematically reviewed the electronic medical records (EMRs) of adult patients diagnosed with solid tumors who developed severe OM during systemic cancer therapy and were subsequently treated with AH between 1 January 2020, and 30 September 2024. Since the primary known clinical indication for AH is the achievement of hemostasis, its therapeutic use in our clinic is specifically directed towards patients who develop hemorrhagic mucositis. In our institutional practice, hemorrhagic mucositis was clinically defined as WHO Grade 3 or 4 OM accompanied by active mucosal surface bleeding, capillary oozing, or hemorrhagic exudate overlying ulcerative lesions. All patients included in this study met this definition at the time of AH initiation. All identified eligible patients received a uniform AH protocol. To minimize selection bias and ensure a homogenous cohort, strict inclusion and exclusion criteria were applied. The inclusion criteria were as follows: (1) age > 18 years, (2) histologically confirmed solid malignancy, (3) receiving active systemic oncological treatment (chemotherapy, targeted therapy or immunotherapy), and (4) development of high-grade OM (WHO Grade 3 or 4). The exclusion criteria were as follows: (1) receipt of concurrent head and neck radiotherapy (to exclude radiation-induced OM), (2) concurrent use of other mucosal healing agents (e.g., glutamine-based therapies, benzydamine), (3) presence of active viral or fungal oral infections prior to AH initiation, and (4) incomplete clinical documentation. Cases were identified by systematically screening the EMR system for all patients with solid tumors who received AH during the study period. To minimize selection bias and ensure transparency, all patients who met the inclusion criteria were consecutively included, with no patients excluded other than those failing to meet the predefined inclusion/exclusion criteria. Following these criteria, from an initial pool of 52 patients identified via EMR search, 43 eligible patients were included in the final analysis (Figure 1).

### 2.2. Therapeutic Administration and Standard Clinical Protocol

AH (Ankaferd Blood Stopper^®^, Ankaferd Health Products Inc., Istanbul, Turkey) is a Turkish Ministry of Health-approved hemostatic agent, available in ampoule form and requiring no special storage conditions. The same standardized formulation was used consistently throughout the study period (2020–2024). The AH mouthwash was administered according to our institutional protocol. The solution was freshly prepared before each application by adding 1 mL of standardized AH extract to 10 mL of boiled and subsequently cooled water. Patients rinsed the oral cavity with the entire freshly prepared solution (11 mL total) for one minute, twice daily, and subsequently expectorated the solution. Administration was scheduled approximately 2 h before meals in the morning and at least 2 h after the evening meal, with no other oral products used within this interval. Treatment was continued until complete mucosal recovery (WHO Grade 0) or clinical stabilization (WHO Grade 1) was achieved, at the treating clinician’s discretion. Treatment adherence was assessed through documentation in the patients’ medical records, including physician notes obtained during routine outpatient visits and hospital admissions, together with tracking of the quantity of AH dispensed to each patient. As per institutional protocol, all patients with Grade 3–4 OM had their systemic anticancer therapy temporarily withheld until mucosal recovery was achieved, in accordance with standard oncological practice. All patients received routine nutritional support during this period, and standard oral hygiene measures, including soft-bristled toothbrushing and bicarbonate mouth rinse, were maintained throughout treatment. No patients in this cohort received concurrent corticosteroid therapy. Hydration was managed as part of standard institutional supportive care. Systemic antibiotic therapy was administered on an as-needed basis when clinically indicated, rather than routinely. No patients received antifungal therapy, as none of the included patients had clinical evidence of oral fungal infection at baseline or during the observation period. Pain management was administered according to a step-up clinical protocol based on the severity of symptoms: paracetamol was utilized for mild-to-moderate pain, whereas opioid analgesics (e.g., tramadol or morphine) were prescribed for severe, refractory pain, administered on an as-needed rather than scheduled basis, with no additional analgesic agents or topical anesthetics used. Analgesic therapy was not newly initiated at the time of AH administration; patients continued their pre-existing as-needed analgesic use according to individual symptom burden.

### 2.3. Clinical Outcomes and Assessment Criteria

The primary endpoint of this study was the clinical response rate, defined as the proportion of patients achieving complete mucosal healing (regression to WHO Grade 0) and the median time to WHO Grade ≤ 1. The secondary endpoints were the reduction in pain intensity and the safety profile of the AH treatment. AH treatment was initiated at the time of clinical presentation with hemorrhagic, Grade 3–4 OM. Despite the retrospective nature of the study, clinical evaluations were highly standardized. As part of our routine institutional inpatient care, the severity of OM and pain intensity were assessed and documented daily by a single attending medical oncologist. The severity of OM was graded using the WHO Oral Mucositis Grading Scale, based on the attending oncologist’s direct clinical assessment WHO grade recorded in the patient’s daily notes, in accordance with the standard WHO criteria. Grade 0 indicated complete recovery, consistent with WHO criteria. Grade 1 was operationally defined as partial recovery for the purposes of this study. Treatment discontinuation was determined at the treating clinician’s discretion, occurring either upon achievement of complete mucosal recovery (Grade 0) or upon clinical stabilization with resolution of active ulceration and hemorrhage (Grade 1), the latter being considered the best clinical response for patients not reaching Grade 0. Pain intensity was evaluated daily using an 11-point Numeric Rating Scale (NRS, 0–10), from baseline (AH initiation) until treatment discontinuation. Adverse events related to AH administration were assessed daily by the senior attending medical oncologist, from AH initiation until treatment discontinuation, through active clinical solicitation of prespecified symptoms (including xerostomia, dysgeusia, oral hypersensitivity reactions, oral dysesthesia, headache, and nausea) using structured institutional questionnaire forms, supplemented by free-text notes in the patient and observation charts. Causality attribution to AH was based on clinical judgment by the treating oncologist, and events were graded according to the Common Terminology Criteria for Adverse Events (CTCAE) version 5.0. The final clinical assessment for each patient corresponded to the day of treatment discontinuation; no fixed maximum observation period was applied, as the observation window for each patient was defined by the duration of AH treatment. No patient was lost to follow-up before completion of AH treatment; all patients were followed until treatment discontinuation.

### 2.4. Statistical Analysis

All analyses were performed in Python 3.12.10 using pandas 2.2.3, NumPy 2.3.5, SciPy 1.16.3, and statsmodels 0.14.6 and SPSS for Windows version 25.0. Categorical variables were summarized as counts and percentages, whereas ordinal and non-normally distributed continuous variables were summarized as medians and interquartile ranges (IQRs). Pre- and post-treatment WHO oral mucositis grades and NRS pain scores were compared using two-sided Wilcoxon signed-rank tests. Signed-rank statistics were standardized using the tie-corrected null variance without continuity correction; effect size was calculated as r = |Z|/sqrt(N), where N was the number of non-missing paired observations. Exact sign-randomization *p* values were used. Median paired changes and 95% confidence intervals (CIs) were estimated using percentile bootstrap resampling (20,000 samples; seed 2026). WHO Grade 0 was not analyzed as a time-to-event endpoint. In a subset of patients, treatment and follow-up were discontinued once Grade 1 was reached, before Grade 0 could be observed; this precludes the assumption of non-informative censoring for the Grade 0 outcome. Findings related to mucosal recovery are therefore reported as follows: time to Grade ≤ 1, the observed proportion of patients achieving Grade 0 (Clopper–Pearson 95% CI), the WHO grade distribution at observation days, and the descriptive time to Grade 0 (median, IQR) among patients who reached this outcome. Time to WHO Grade ≤ 1 for baseline Grade 3 versus Grade 4 was compared using an exact permutation Mann–Whitney U test accounting for tied ranks; rank-biserial correlation and a Hodges–Lehmann shift estimate were reported, with percentile-bootstrap CIs. Recovery proportions were accompanied by Clopper–Pearson exact 95% CIs. Fisher’s exact test was used for sparse 2 × 2 tables, and 100,000-label Monte Carlo permutation tests were used for sparse larger contingency tables. Analyses used available cases without imputation (Supplementary Table S4). All tests were two-sided with alpha = 0.05. Exploratory analyses were not adjusted for multiplicity and should therefore be interpreted cautiously. No formal a priori sample-size calculation was performed because of the retrospective design; all consecutive eligible patients treated during the predefined study period who met the inclusion and exclusion criteria were included.

### 2.5. Ethical Considerations

The study was conducted in accordance with the principles outlined in the Declaration of Helsinki. Retrospective ethical approval was granted by the Non-Interventional Clinical Research Ethics Committee of Adnan Menderes University Faculty of Medicine (Approval Date: 12 December 2024; Approval No: 2024/189). Given the retrospective observational design and the use of de-identified data, the requirement for informed consent was waived by the institutional review board.

## 3. Results

### 3.1. Patient Characteristics

A total of 43 patients were included in the final analysis. The median age of the cohort was 68 years (IQR: 58–71.5), with 55.8% (*n* = 24) aged ≥65 years, and a male predominance (60.5%, *n* = 26). Regarding systemic oncological treatments, 65.1% received chemotherapy alone; notably, no patients received concurrent head and neck radiotherapy, consistent with the exclusion criteria. At baseline, the severity of OM was assessed as Grade 3 in 81.4% (*n* = 35) and Grade 4 in 18.6% (*n* = 8) of the patients (Table 1). Detailed patient diagnoses and oncological treatment protocols are provided in Supplementary Table S1.

### 3.2. Mucosal Healing Outcomes During Ankaferd Hemostat Use

**Figure 2:**
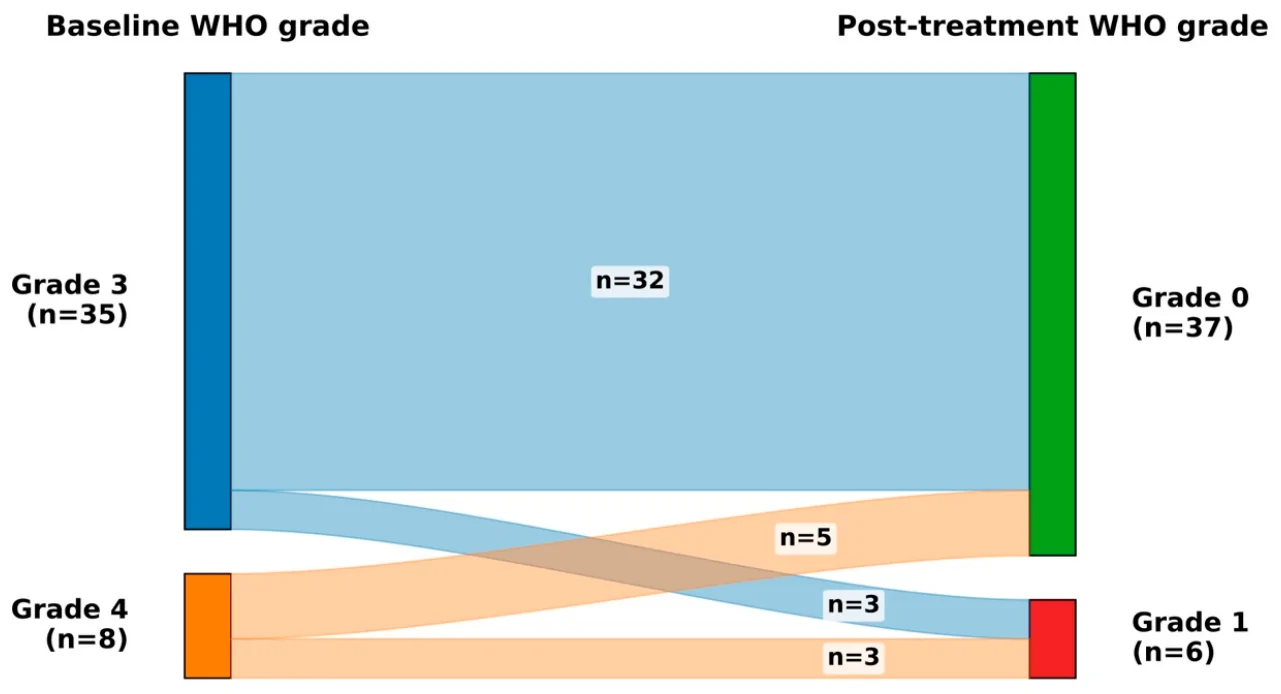
Individual transitions in WHO oral mucositis grade from baseline to post-treatment assessment (N = 43). Band width is proportional to the number of patients undergoing each grade transition. Among 35 patients with baseline Grade 3 mucositis, 32 transitioned to Grade 0 and three to Grade 1; among eight with baseline Grade 4, five transitioned to Grade 0 and three to Grade 1. A within-patient reduction was observed (exact Wilcoxon signed-rank W = 0, Z = −6.125, *p* < 0.001, r = 0.934).

Following AH use, complete mucosal recovery (regression to WHO Grade 0) was observed in 37/43 patients (86.0%; exact 95% CI 72.1–94.7%). Among patients with baseline Grade 3 OM, 32/35 achieved complete recovery (91.4%; exact 95% CI 76.9–98.2%), whereas 3/35 (8.6%) improved to Grade 1. Among patients with baseline Grade 4 OM, 5/8 achieved complete recovery (62.5%; exact 95% CI 24.5–91.5%), whereas 3/8 (37.5%) improved to Grade 1. The median WHO mucositis grade decreased from 3 (IQR 3–3) at baseline to 0 (IQR 0–0) after treatment (exact Wilcoxon signed-rank W = 0, Z = −6.125, *p* < 0.001, r = 0.934) (Figure 2). The median paired improvement was 3 grades (percentile-bootstrap 95% CI 3–3). This analysis demonstrates a substantial within-patient reduction in mucositis grade during the treatment period; however, in the absence of a control group, the observed change does not by itself establish treatment efficacy. The difference in complete recovery rates between baseline Grade 3 and Grade 4 patients was not statistically significant (Fisher’s exact OR = 6.01, 95% CI 0.63–59.45; *p* = 0.067), likely reflecting limited precision in the small Grade 4 subgroup.

Exploratory analyses did not identify statistically detectable associations between complete mucosal recovery and age (OR per 10-year increase 1.29, 95% CI 0.66–2.52; *p* = 0.456), sex (female vs. male conditional OR 3.58, exact 95% CI 0.44–44.57; Fisher’s exact *p* = 0.193), or treatment modality (100,000-label permutation *p* = 0.248) (Supplementary Tables S2 and S3). The sparse-table permutation analysis for tumor type yielded an unadjusted *p* = 0.0452; however, 10 of the 15 tumor categories contained no more than two patients and only six incomplete recoveries occurred. Accordingly, this finding was considered unstable and exploratory and was not interpreted as evidence of a tumor-specific association. Confidence intervals were wide, and the study was not powered to exclude clinically meaningful associations.

### 3.3. Time to Recovery and Pain Management

With respect to the recovery-related endpoints, all 43 patients achieved WHO Grade ≤ 1, with a median time of 3 days (IQR 2–3; range 2–5) overall. Time to this outcome was numerically similar between subgroups (3 days [IQR 2–3] for Grade 3 versus 3 days [IQR 3–4.25] for Grade 4) but differed significantly in the underlying rank distribution (exact permutation Mann–Whitney U test, U = 205.0, *p* = 0.0233; rank-biserial r = 0.464, 95% CI 0.064–0.800; Hodges–Lehmann shift 1 day, 95% CI 0–2), reflecting a wider spread and later upper-range values among Grade 4 patients (Table 2). The cross-sectional distribution of WHO grades at fixed post-baseline days is presented descriptively in Supplementary Table S5 and Supplementary Figure S1.

Complete mucosal recovery (WHO Grade 0) was observed in 37/43 patients overall (86.0%; exact 95% CI 72.1–94.7%), compared with 32/35 (91.4%) among Grade 3 patients and 5/8 (62.5%) among Grade 4 patients; this difference was not statistically significant (Fisher’s exact test, *p* = 0.067), likely reflecting limited precision in the small Grade 4 subgroup (Table 2). Among the 37 patients who achieved Grade 0, the descriptive median time to this outcome was 4 days (IQR 4–5; range 3–6) overall, 4 days (IQR 4–4.5) among Grade 3 patients (*n* = 32), and 5 days (IQR 5–5) among Grade 4 patients (*n* = 5). As this estimate is conditional on having reached Grade 0, an outcome not attained by all patients, no formal inferential comparison between subgroups was undertaken; these values should therefore be interpreted as descriptive rather than confirmatory. Given the limited size of the Grade 4 subgroup (*n* = 8), these estimates warrant cautious interpretation.

**Figure 3:**
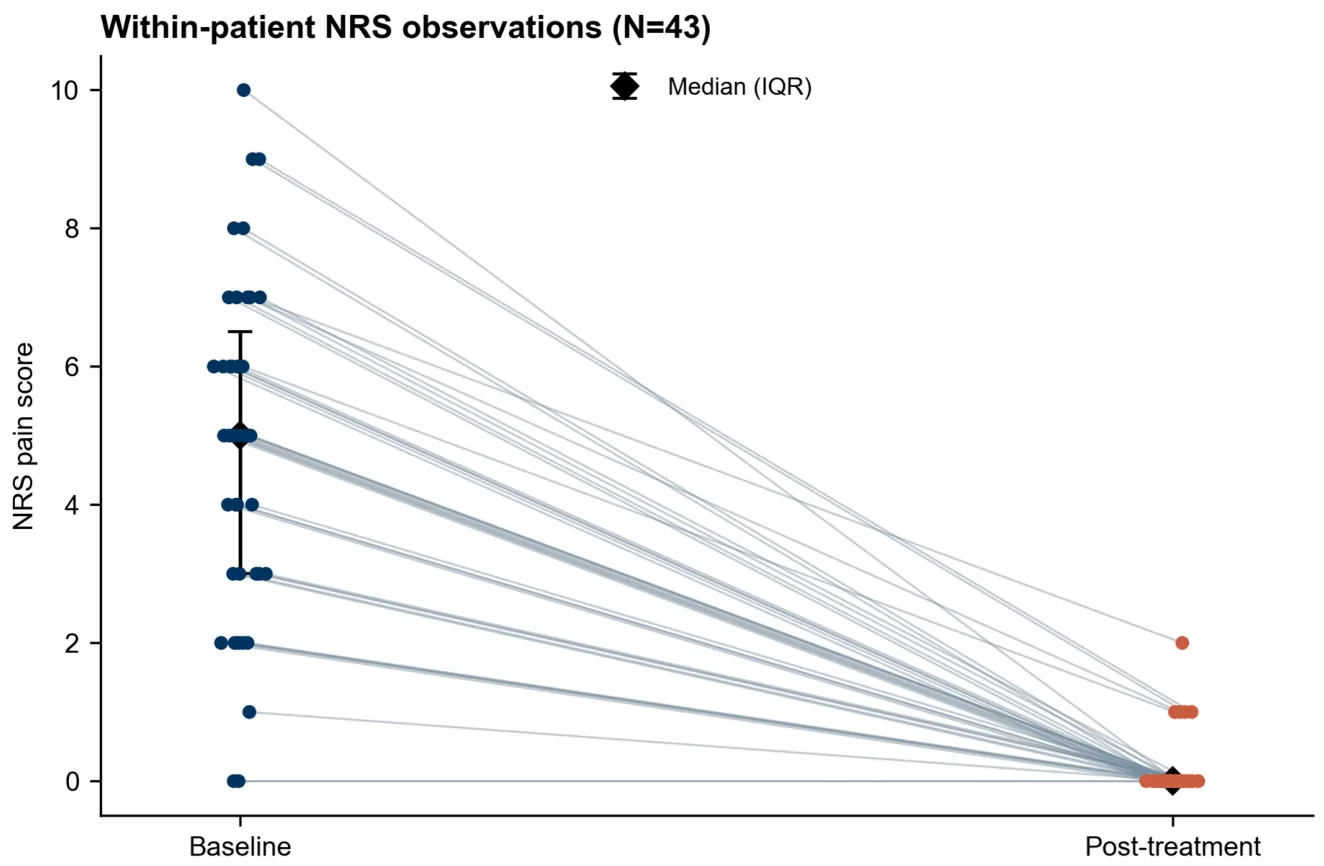
Paired NRS pain scores at baseline and post-treatment assessment (N = 43). Each line represents one patient, with paired observations connected between baseline and post-treatment measurements; circles indicate individual NRS scores. Diamonds indicate median values and vertical bars represent the interquartile range. Median NRS score decreased from 5 (IQR 3–6.5) at baseline to 0 (IQR 0–0) after treatment (exact Wilcoxon signed-rank W = 0, Z = −5.598, *p* < 0.001, r = 0.854). The analysis describes within-patient change during the treatment period.

Median NRS pain score decreased from 5 (IQR 3–6.5) at baseline to 0 (IQR 0–0) after treatment (exact Wilcoxon signed-rank W = 0, Z = −5.598, *p* < 0.001, r = 0.854). The median paired improvement was 5 points (percentile-bootstrap 95% CI 4–5). This substantial within-patient reduction occurred during a period in which concurrent systemic analgesics could be administered; therefore, the observed pain reduction cannot be attributed exclusively to AH (Figure 3).

### 3.4. Safety Profile

No Grade ≥ 2 adverse events or serious unexpected adverse reactions were reported with the use of AH mouthwash in this cohort; however, the retrospective design and limited sample size preclude definitive conclusions regarding safety, particularly for uncommon or delayed adverse events. Only one patient developed Grade 1 xerostomia, which was transient and did not necessitate treatment interruption. Furthermore, no local or systemic toxicities, such as dysgeusia (taste alterations), severe hypersensitivity reactions, oral dysesthesia, headache or nausea, were observed.

## 4. Discussion

In this study, AH mouthwash administration was associated with a Grade 0 mucosal recovery rate of 86.0% (37/43; 95% CI 72.1–94.7%) and a median time to recovery of four days (IQR 4–5) in patients with severe (WHO Grade 3 and 4) OM. To our knowledge, this is among the first clinical investigations to evaluate the therapeutic use of AH explicitly in a cohort of patients with solid tumors, directly addressing a major unmet clinical need in oncological practice. Given the scarcity of studies specifically addressing this severe (Grade 3–4) subgroup, we drew on both AH studies in other populations and studies of other agents in more comparable populations, bearing in mind differences in design and outcome definitions. For instance, in a phase III randomized clinical trial evaluating a mouthwash (Plantago major, chlorhexidine, and sodium bicarbonate), Cabrera-Jaime et al. reported a complete recovery rate of 68% and a mean healing time of 7 days, in a cohort in which 86% of patients presented with only Grade 2 mucositis [16]. Direct comparison with our cohort is not possible given the differences in baseline severity, administration, follow-up duration, and outcome definitions; nevertheless, the shorter recovery time observed in our more severe (Grade 3–4) population is noteworthy and warrants confirmation in prospective comparative studies. In a separate, randomized trial of 200 patients, Dodd et al. reported a mean time to resolution of chemotherapy-induced oral mucositis of approximately 7 days across different standard oral care regimens [17]. Similarly, while Erdem and Güngörmüş reported rapid OM healing times utilizing royal jelly, their study population was heavily skewed towards lower-grade toxicities, with Grade 3 OM comprising only 7.6% of the cohort [18]. In the control arm of that same study, Grade 3 OM required approximately 10 days to resolve [18]. By comparison, our analysis focused exclusively on a severe patient population, encompassing only WHO Grade 3 and 4 mucositis, and observed a median recovery time of 4 days. This median time to recovery was also numerically shorter than in earlier reports of AH use in OM. Atay et al. reported a median recovery time of 6.6 days (min-max: 3–10) in patients receiving AH up to four times daily [14]. The accelerated healing dynamics observed in our cohort may stem from the pathophysiological response of highly hemorrhagic mucosa to AH. Upon application, AH rapidly forms an encapsulated protein network that ensures effective hemostasis. Concurrently, this structural matrix suppresses the local release of inflammatory mediators and accelerates tissue regeneration via fibroblast proliferation [11,12,13].

Beyond its therapeutic application, the role of AH in the prophylaxis of OM warrants consideration. A study by Patıroğlu et al. demonstrated that integrating AH into standard oral care significantly mitigated the severity of OM in a prophylactic setting [15]. Furthermore, in a cohort of colorectal cancer patients receiving FOLFOX chemotherapy, AH provided a significantly superior prophylactic benefit against OM development when compared to standard bicarbonate rinses [10]. More recently, Begendi and Duran reported, in a retrospective comparative study of patients with hematologic malignancies, that AH and benzydamine hydrochloride were both associated with significantly lower rates of chemotherapy-induced OM than sodium bicarbonate when used prophylactically, further supporting a potential preventive role for AH [19]. Although our study was designed to evaluate the therapeutic management of high-grade OM, these comparative literature findings suggest that AH may offer a dual clinical role: a prophylactic effect in preventing OM onset, and a possible therapeutic effect in managing established mucosal breakdown.

Effective pain management is critical in OM to prevent malnutrition and treatment interruptions [1,20]. To date, no studies in the oncological literature have specifically investigated the analgesic effect of AH in OM patients. However, our finding of a marked reduction in NRS scores (median decrease from 5 to 0) parallels the results of Yaman et al., who reported favorable short and long-term pain control with AH in pediatric pulpotomy models [21]. This observed reduction in pain may be related to AH’s anti-inflammatory properties combined with its physical ability to coat and shield exposed subepithelial nerve endings from oral stimuli [11]. Nevertheless, as patients in our study received concurrent systemic analgesics per clinical protocols when necessary, attributing the observed pain palliation exclusively to AH requires cautious interpretation.

Regarding tolerability, no Grade ≥ 2 adverse events were observed in this cohort, consistent with the previous literature describing AH as a well-tolerated hemostatic agent [10,14,22]. While previous studies noted mild, clinically insignificant adverse events such as a temporary metallic taste or minor gastrointestinal symptoms [14,22]. Our cohort reported only a single case of Grade 1 dry mouth. However, the small sample size and retrospective design limit definitive conclusions regarding rare or delayed adverse effects.

This study has several limitations. It was retrospective, single-center, single-arm, and lacked a control group, so spontaneous improvement in OM cannot be excluded and the results are descriptive rather than comparative. The sample size was small (*n* = 43), limiting power, especially for the Grade 4 subgroup. Only hemorrhagic high-grade OM was included, so findings may not apply to non-hemorrhagic or lower-grade mucositis. Our findings, derived from solid tumor patients, may not be generalizable to hematologic malignancies, radiotherapy-induced mucositis, or stem-cell transplant recipients, given their distinct mucositis pathophysiology. The cohort was heterogeneous in tumor type and systemic therapy category, and potential confounders (timing of OM relative to cancer treatment, hematologic and nutritional status, diabetes, smoking, and oral hygiene) were not systematically recorded or adjusted for. OM grade and adverse events were assessed daily by a single unblinded oncologist without inter-rater reliability testing; although the WHO scale is standardized, some observer bias is possible, and mild-to-moderate adverse events may have been underreported. Pain scores were recorded while patients continued as-needed analgesics, so pain reduction cannot be attributed to AH alone. Patients who discontinuation at Grade 1 without reaching Grade 0 had no further follow-up, so their later course is unknown. These findings are therefore hypothesis-generating, and prospective randomized controlled studies are needed for confirmation.

## 5. Conclusions

In conclusion, this retrospective study suggests that AH mouthwash was not associated with severe adverse events and may represent an easily implementable supportive care option for managing severe OM in solid tumor patients. The clinical observations of rapid mucosal recovery and substantial pain palliation indicate a favorable safety and tolerability profile, supporting its potential role in alleviating the acute symptomatic burden of high-grade OM. While these real-world findings are promising, they should be interpreted in light of the retrospective design and its inherent limitations. Prospective, randomized controlled trials are warranted to validate these observations and to more definitively characterize the efficacy of AH before its integration into routine supportive oncology care can be recommended.

## Figures and Tables

**Table 1:** Baseline demographic and clinical characteristics of the study population.

Characteristic	N = 43
**Age, years, median (IQR)**	68 (58–71.5)
**Sex, *n* (%)**	
Female	17 (39.5)
Male	26 (60.5)
**Tumor type, *n* (%)**	
Colon cancer	10 (23.3)
Non-Small Cell Lung cancer	8 (18.6)
Breast cancer	6 (14.0)
Small Cell Lung cancer	3 (7.0)
Stomach cancer	3 (7.0)
Pancreatic cancer	2 (4.7)
Other *	8 (18.6)
**Systemic treatment, *n* (%)**	
Chemotherapy alone	28 (65.1)
Targeted therapy alone	6 (14.0)
Chemotherapy plus targeted therapy	7 (16.3)
Immunotherapy alone	1 (2.3)
Antibody–drug conjugate	1 (2.3)
**Baseline WHO Oral Mucositis Grade, *n* (%)**	
Grade 3	35 (81.4)
Grade 4	8 (18.6)
**Baseline NRS pain score, median (IQR)**	5 (3–6.5)

Values are median (IQR), *n* (%), or range as indicated. * Other includes testicular, endometrial, pancreatic neuroendocrine, prostate, biliary tract, malignant melanoma, brain tumor, and cervical cancer (*n* = 1 each). N: Number of patients, WHO: World Health Organization.

**Table 2:** Clinical outcomes according to baseline WHO Oral Mucositis Grading Scale.

Outcome	Overall (N = 43)	Baseline Grade 3 (*n* = 35)	Baseline Grade 4 (*n* = 8)
Complete recovery, *n*/N (%) [exact 95% CI]	37/43 (86.0%) [72.1–94.7]	32/35 (91.4%) [76.9–98.2]	5/8 (62.5%) [24.5–91.5]
Partial recovery, *n*/N (%) [exact 95% CI]	6/43 (14.0%) [5.3–27.9]	3/35 (8.6%) [1.8–23.1]	3/8 (37.5%) [8.5–75.5]
Time to WHO Grade ≤ 1, days, median (IQR)	3 (2–3)	3 (2–3)	3 (3–4.25)
Time to WHO Grade ≤ 1, range	2–5	2–4	2–5
Time to WHO Grade 0 among patients achieving Grade 0, days, median (IQR)	4 (4–5); *n* = 37	4 (4–4.5); *n* = 32	5 (5–5); *n* = 5
Time to WHO Grade 0 among patients achieving Grade 0, range	3–6	3–6	3–6

Between-group comparisons were performed as follows: complete and partial recovery rates were compared using Fisher’s exact test (*p* = 0.067). Time to WHO Grade ≤ 1 was compared using an exact permutation Mann–Whitney U test (U = 205.0; *p* = 0.0233), with rank-biserial r = 0.464 (95% CI 0.064–0.800) and a Hodges–Lehmann shift of 1 day (95% CI 0–2). Time to WHO Grade 0 among patients achieving Grade 0 was presented descriptively and was not compared because this analysis conditions on event occurrence. Complete/partial recovery rows use the final clinical outcome field. Their exact confidence intervals are Clopper–Pearson intervals. Time to WHO Grade ≤ 1 was defined as days from baseline to the first recorded WHO grade of 1 or 0. All 43 patients experienced this endpoint; no censoring occurred. Time to WHO Grade 0 was defined as days from baseline to the first recorded WHO Grade 0. NE, not estimable; HL, Hodges–Lehmann; KM, Kaplan–Meier. The Cox hazard ratio was not retained because the proportional-hazards assumption was violated (Schoenfeld-residual test *p* = 0.002).

## Data Availability

The data sets and analyses from the study can be obtained from the corresponding author upon reasonable request. The data are not publicly available due to privacy and ethical restrictions.

## Supplementary Materials

The following supporting information can be downloaded at https://www.mdpi.com/article/10.3390/healthcare14183005/s1, Table S1: The diagnoses and oncological treatment protocols of the patients. Table S2. Clinical outcomes stratified by systemic anticancer treatment category. Table S3. Exploratory associations with complete mucosal recovery. Table S4. Missing data for variables included in the statistical analyses. Table S5. Distribution of WHO oral mucositis grades at follow-up days. Supplementary Figure S1. Distribution of WHO oral mucositis grades at selected follow-up days (Days 0, 1, 3, 5, and 7).

## Abbreviations

The following abbreviations are used in this manuscript:

AH	Ankaferd Hemostat
ANOVA	Analysis of Variance
CTCAE	Common Terminology Criteria for Adverse Events
EMR	Electronic Medical Record
ESMO	European Society for Medical Oncology
IL-6	Interleukin-6
IQR	Interquartile Range
ISOO	International Society of Oral Oncology
MASCC	Multinational Association of Supportive Care in Cancer
NRS	Numeric Rating Scale
OM	Oral Mucositis
ROS	Reactive Oxygen Species
SPSS	Statistical Package for the Social Sciences
TNF-α	Tumor Necrosis Factor Alpha
WHO	World Health Organization
