# Supplementary material for: Clinical Outcomes of Ankaferd Hemostat Mouthwash for High-Grade Oral Mucositis During Systemic Anticancer Therapy: A Single-Center, Retrospective, Single-Arm Cohort Study
Source: Healthcare (Basel). 2026 Sep 14;14(18):3005. doi: 10.3390/healthcare14183005 (PMC13606083; doi:10.3390/healthcare14183005)

Supplementary Figure S1. Distribution of WHO oral mucositis grades at selected follow-up days (Days 0, 1, 3, 5, and 7).

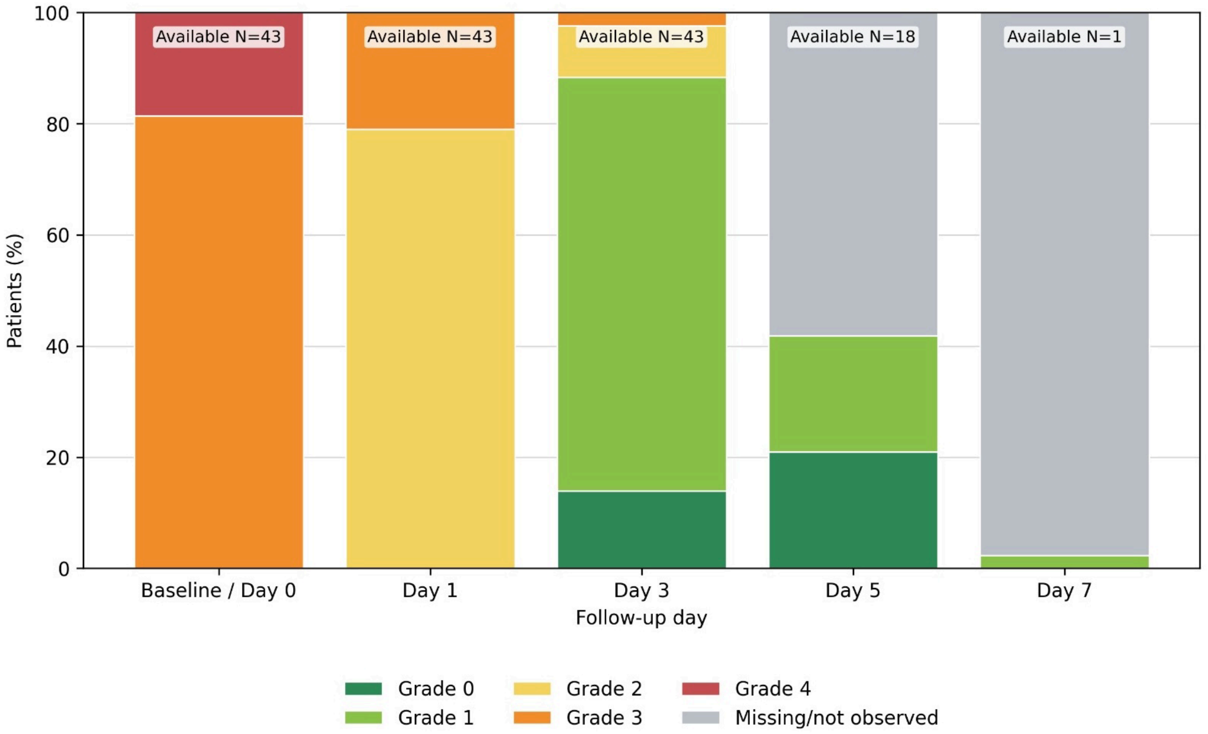

Supplement: Supplementary file 1 [file healthcare-14-03005-s001.zip › Supplementary Figure S1.pdf]
