# Supplementary material for: Clinical Outcomes of Ankaferd Hemostat Mouthwash for High-Grade Oral Mucositis During Systemic Anticancer Therapy: A Single-Center, Retrospective, Single-Arm Cohort Study
Source: Healthcare (Basel). 2026 Sep 14;14(18):3005. doi: 10.3390/healthcare14183005 (PMC13606083; doi:10.3390/healthcare14183005)
Supplement: Supplementary file 1 [file healthcare-14-03005-s001.zip › Supplementary Table S1.pdf]

Supplementary Table S1: The diagnoses and oncological treatment protocols of the patients

Patient no	Type of disease	Treatment regimens
1	Breast cancer	Paclitaxel
2	Non-small cell lung cancer	Carboplatin + paclitaxel
3	Colon cancer	Capecitabine
4	Colon cancer	Regorafenib
5	Colon cancer	Folfiri
6	Renal cell cancer	Nivolumab + ipilumumab
7	Colon cancer	Folfox + panitumumab
8	Small cell lung cancer	Carboplatin + paclitaxel
9	Testicular cancer	BEP
10	Non-small cell lung cancer	Cisplatin + pemetrexed
11	Breast cancer	Cdk4/6 inhibitor
12	Endometrium cancer	Carboplatin + paclitaxel
13	Pancreatic neuroencrine carcinoma	Everolimus
14	Renal cell cancer	Cabozantinib
15	Pancreatic cancer	Capecitabine + docetaxel + gemcitabine
16	Breast cancer	EC
17	Prostate cancer	Docetaxel
18	Breast cancer	Docetaxel + trastuzumab + pertuzumab
19	Pancreatic cancer	Gemcitabine
20	Small cell lung cancer	Carboplatin + etoposide
21	Biliary tract cancer	Capecitabine
22	Malignant melanoma	Paclitaxel
23	Brain tumor	Irinotecan + bevacizumab
24	Non-small cell lung cancer	Etoposide
25	Non-small cell lung cancer	Carboplatin + paclitaxel
26	Colon cancer	Folfiri + bevacizumab
27	Non-small cell lung cancer	Cisplatin + pemetrexed
28	Colon cancer	Regorofenib
29	Renal cell cancer	Cabozantinib
30	Small cell lung cancer	Cisplatin + etoposide
31	Colon cancer	Folfox

32	Non-small cell lung cancer	Gemcitabine + carboplatin
33	Non-small cell lung cancer	Carboplatin + paclitaxel
34	Stomach cancer	Folfox
35	Colon cancer	Folfox + bevacizumab
36	Cervix cancer	Cisplatin
37	Colon cancer	Folfox + bevacizumab
38	Non-small cell lung cancer	Carboplatin + paclitaxel
39	Colon cancer	Folfiri + bevacizumab
40	Stomach cancer	Folfox
41	Breast cancer	Sacitumub govitekan
42	Stomach cancer	Folfox
43	Breast cancer	AC

BEP: Bleomisin etoposid cisplatin, EC: Epirubicin + cyclophosphamide, AC: Doxorubicin + cyclophosphamide, Folfox: Fluorouracil + leucovorin + oxaliplatin, Folfiri: Fluorouracil + leucovorin + irinotecan
