# Supplementary material for: Clinical Outcomes of Ankaferd Hemostat Mouthwash for High-Grade Oral Mucositis During Systemic Anticancer Therapy: A Single-Center, Retrospective, Single-Arm Cohort Study
Source: Healthcare (Basel). 2026 Sep 14;14(18):3005. doi: 10.3390/healthcare14183005 (PMC13606083; doi:10.3390/healthcare14183005)
Supplement: Supplementary file 1 [file healthcare-14-03005-s001.zip › Supplementary Table S2.pdf]

Supplementary Table S2. Clinical outcomes stratified by systemic anticancer treatment category.

Treatment category	n	Grade 0, n (%)	Grade 1, n (%)	Recovery time, days*	Baseline NRS pain*	Post-Treatment NRS pain*
Chemotherapy only	28	24 (85.7)	4 (14.3)	4 (4–5)	5.5 (2.75–7.0)	0 (0–0)
Chemotherapy + targeted therapy	7	6 (85.7)	1 (14.3)	4 (4–4)	4.0 (3.0–4.0)	0 (0–0)
Targeted therapy only	6	6 (100.0)	0 (0.0)	4 (3–5)	5.0 (3.5–5.75)	0 (0–0)
Immunotherapy only	1	1 (100.0)	0 (0.0)	4	5	0
Antibody–drug conjugate	1	0 (0.0)	1 (100.0)	5	8	0
**Overall cohort**	**43**	**37 (86.0)**	**6 (14.0)**	**4 (4–5)**	**5.0 (3.0–6.5)**	**0 (0–0)**

Values are n (%) for categorical variables. *For groups with n ≥ 5, recovery time and NRS pain scores are reported as median (interquartile range); for the Immunotherapy-only and Antibody–drug conjugate groups (n = 1 each), the single patient's observed value is reported instead of a median/IQR. NRS: Numeric Rating Scale (0–10). Given the small number of patients in some treatment categories (particularly n = 1 for immunotherapy and antibody–drug conjugate groups), these estimates are descriptive only and should be interpreted with caution. An exploratory global permutation test found no statistically detectable association between treatment modality and complete mucosal recovery (100,000-label permutation p = 0.248); however, this test is underpowered given the sample size and marked imbalance in group sizes, and should not be interpreted as evidence of no true difference between categories.
