# Supplementary material for: Clinical Outcomes of Ankaferd Hemostat Mouthwash for High-Grade Oral Mucositis During Systemic Anticancer Therapy: A Single-Center, Retrospective, Single-Arm Cohort Study
Source: Healthcare (Basel). 2026 Sep 14;14(18):3005. doi: 10.3390/healthcare14183005 (PMC13606083; doi:10.3390/healthcare14183005)
Supplement: Supplementary file 1 [file healthcare-14-03005-s001.zip › Supplementary Table S3.pdf]

Supplementary Table S3. Exploratory associations with complete mucosal recovery

Variable / category	Complete recovery	Partial recovery	Effect estimate (95% CI)	Test	p-value
**Age, years, median (IQR)**	67 (59–71)	69.5 (47–71.75)	OR per 10-year increase = 1.29 (0.66–2.52)	Univariable logistic regression	0.456
**Sex**					
**Female**	13/17 (76.5%)	4/17 (23.5%)	OR = 3.58 (0.44–44.57)†	Fisher's exact test	0.193
**Male**	24/26 (92.3%)	2/26 (7.7%)	Reference		
**Tumor type**			**Cramér's V = 0.756 (bootstrap 95% CI 0.532–1.000)**	100,000-permutation sparse R×2 test	**0.0452‡**
**Biliary tract cancer**	1/1 (100%)	0/1 (0%)	—	—	—
**Brain tumor**	0/1 (0%)	1/1 (100%)	—	—	—
**Breast cancer**	5/6 (83.3%)	1/6 (16.7%)	—	—	—
**Cervical cancer**	0/1 (0%)	1/1 (100%)	—	—	—
**Colon cancer**	10/10 (100%)	0/10 (0%)	—	—	—
**Endometrial cancer**	0/1 (0%)	1/1 (100%)	—	—	—
**Malignant melanoma**	1/1 (100%)	0/1 (0%)	—	—	—
**Non-small cell lung cancer**	7/8 (87.5%)	1/8 (12.5%)	—	—	—
**Pancreatic cancer**	1/2 (50.0%)	1/2 (50.0%)	—	—	—
**Pancreatic neuroendocrine carcinoma**	1/1 (100%)	0/1 (0%)	—	—	—
**Prostate cancer**	1/1 (100%)	0/1 (0%)	—	—	—
**Renal cell cancer**	3/3 (100%)	0/3 (0%)	—	—	—
**Small cell lung cancer**	3/3 (100%)	0/3 (0%)	—	—	—
**Stomach cancer**	3/3 (100%)	0/3 (0%)	—	—	—
**Testicular cancer**	1/1 (100%)	0/1 (0%)	—	—	—
**Treatment modality**			**Cramér's V = 0.412 (bootstrap 95% CI 0.131–0.741)**	100,000-permutation sparse R×2 test	0.248
**Antibody–drug conjugate**	0/1 (0%)	1/1 (100%)	—	—	—
**Chemotherapy + targeted therapy**	6/7 (85.7%)	1/7 (14.3%)	—	—	—

**Chemotherapy only**	24/28 (85.7%)	4/28 (14.3%)	—	—	—
**Immunotherapy only**	1/1 (100%)	0/1 (0%)	—	—	—
**Targeted therapy only**	6/6 (100%)	0/6 (0%)	—	—	—

CI, confidence interval; IQR, interquartile range; OR, odds ratio.

† OR represents the conditional maximum-likelihood odds ratio for female versus male patients.

‡ The tumor-type analysis was exploratory and unadjusted for multiplicity. Tumor categories were not collapsed or reclassified. Because 10 of the 15 tumor categories contained ≤2 patients and only six incomplete recoveries occurred, the global result is highly sensitive to sparse cells and should not be interpreted as evidence of a specific tumor-type association.

All analyses were exploratory. No adjustment for multiple comparisons was applied.
