# Supplementary material for: Clinical Outcomes of Ankaferd Hemostat Mouthwash for High-Grade Oral Mucositis During Systemic Anticancer Therapy: A Single-Center, Retrospective, Single-Arm Cohort Study
Source: Healthcare (Basel). 2026 Sep 14;14(18):3005. doi: 10.3390/healthcare14183005 (PMC13606083; doi:10.3390/healthcare14183005)
Supplement: Supplementary file 1 [file healthcare-14-03005-s001.zip › Supplementary Table S4.pdf]

Supplementary Table S4. Missing data for variables included in the statistical analyses

Variable	Total N	Available, n	Missing, n	Missing, %
**Age**	43	43	0	0.0
**Sex**	43	43	0	0.0
**Baseline WHO grade**	43	43	0	0.0
**Post-treatment WHO grade**	43	43	0	0.0
**Tumor type**	43	43	0	0.0
**Oncological Treatment modality**	43	43	0	0.0
**Oncological Treatment regimen**	43	43	0	0.0
**Baseline NRS**	43	43	0	0.0
**Post-treatment NRS**	43	43	0	0.0
**Adverse event presence**	43	43	0	0.0
**Adverse event type**	43	43	0	0.0
**Adverse event severity**	43	43	0	0.0
**Recovery/follow-up time**	43	43	0	0.0

NRS, Numeric Rating Scale (for pain); WHO, World Health Organization.

Note: No missing observations were identified for variables included in the statistical analyses; therefore, no imputation was performed. Analyses were conducted using available cases. Non-analytic administrative or documentation fields were excluded from the missing-data summary.
