# Supplementary material for: Clinical Outcomes of Ankaferd Hemostat Mouthwash for High-Grade Oral Mucositis During Systemic Anticancer Therapy: A Single-Center, Retrospective, Single-Arm Cohort Study
Source: Healthcare (Basel). 2026 Sep 14;14(18):3005. doi: 10.3390/healthcare14183005 (PMC13606083; doi:10.3390/healthcare14183005)
Supplement: Supplementary file 1 [file healthcare-14-03005-s001.zip › Supplementary Table S5.pdf]

Supplementary Table S5. Distribution of WHO oral mucositis grades at follow-up days

Time point	Available N	Missing n	Grade 0, n (%)	Grade 1, n (%)	Grade 2, n (%)	Grade 3, n (%)	Grade 4, n (%)
**Baseline / Day 0**	43	0	0 (0.0)	0 (0.0)	0 (0.0)	35 (81.4)	8 (18.6)
**Day 1**	43	0	0 (0.0)	0 (0.0)	34 (79.1)	9 (20.9)	0 (0.0)
**Day 3**	43	0	6 (14.0)	32 (74.4)	4 (9.3)	1 (2.3)	0 (0.0)
**Day 5**	18	25	9 (50.0)	9 (50.0)	0 (0.0)	0 (0.0)	0 (0.0)
**Day 7**	1	42	0 (0.0)	1 (100.0)	0 (0.0)	0 (0.0)	0 (0.0)

Percentages use the available N at each time point as the denominator. Missing daily observations were not imputed, interpolated, or carried forward.

Because daily recording frequently stopped after recovery or final classification, the Day 5 and Day 7 distributions describe only the observed subsets (N=18 and N=1, respectively) and should not be interpreted as complete-cohort distributions.
